# A family of ionic supersalts with covalent-like directionality and unconventional multiferroicity

**DOI:** 10.1038/s41467-021-21597-3

**Published:** 2021-02-26

**Authors:** Yaxin Gao, Menghao Wu, Puru Jena

**Affiliations:** 1 School of Physics and Wuhan National High Magnetic Field Center, Huazhong University of Science and Technology, Wuhan, Hubei China; 2 Department of Physics, Virginia Commonwealth University, Richmond, VA USA

**Keywords:** Structure prediction, Atomistic models

## Abstract

Ionic crystals composed of elemental ions such as NaCl are non-polar due to directionless ionic bonding interactions. Here, we show that these can develop polarity by changing their building blocks from elemental ions to superalkali and superhalogen cluster-ions, which mimic the chemistry of alkali and halogen atoms, respectively. Due to the non-spherical geometries of these cluster ions, corresponding supersalts form anisotropic polar structures with ionic bonding, yet covalent-like directionality, akin to sp^3^ hybridized systems. Using density functional theory and extensive structure searches, we predict a series of stable ferroelectric/ferroelastic supersalts, PnH_4_MX_4_ (Pn = N, P; M = B, Al, Fe; X = Cl, Br) composed of superalkali PnH_4_ and superhalogen MX_4_ ions. Unlike traditional ferroelectric/ferroelastic materials, the cluster-ion based supersalts possess ultra-low switching barrier and can endure large ion displacements and reversible strain. In particular, PH_4_FeBr_4_ exhibits triferroic coupling of ferroelectricity, ferroelasticity, and antiferromagnetism with controllable spin directions via either ferroelastic or 90-degree ferroelectric switching.

## Abstract (web summary)

Binary ionic crystals, such as NaCl, are non-polar due to directionless ionic bonding interactions. Here, the authors show that polarity can be developed by using superalkali/superhalogen ions as building blocks, leading to ionic supersalts with ferroelectricity, ferroelasticity or even triferroicity.

## Introduction

Ionic crystals composed of elemental ions are nonpolar due to the directionless feature associated with ionic bonding. In this paper we propose a paradigm shift in material synthesis where ionic crystals can be designed to exhibit directional bonding and lead to materials with long ion-displacement ferroelectricity, high-strain ferroelasticity, and triferroic couplings. The key is to change the building blocks of ionic crystals from elemental ions to cluster ions. This concept was introduced by Khanna and Jena more than 25 years ago when the authors showed that superatomic^1–3^ clusters with appropriate size and composition can be designed to mimic the properties of atoms and, when assembled, could form materials with unique properties. Superhalogens and superalkalis proposed by Gutsev and Boldyrev^4–6^ much earlier belong to a sub-group of these superatoms. Superhalogens have electron affinities (EAs) that are larger than those of halogens (~3.6 eV for Cl) while superalkalis^7–10^ have ionization potentials (IPs) that are smaller than those of alkalies (~3.9 eV for Cs). Prominent examples include superhalogens with formula unit AX_*k*+1_ where *k* the valence of atom A and X is a halogen atom (e.g., BX_4_, AlX_4_, SiX_5_ and PX_6_), and superalkalis with formula unit M_*k*+1_L, where M is an alkali atom and *k* is the formal valence of the electronegative atom L (e.g., Li_3_O, Li_4_N). The former can be used for oxidation of counterpart systems with high IPs and production of organic superconductors^11^, while the latter can find applications in the synthesis of charge-transfer salts where the anions are formed by the species with low EAs. Giri et al. have shown that superalkalis and superhalogens can be combined together to form a new class of supersalts where both the ions retain their structural and compositional identity. Supersalts with tailored properties^12,13^, superbases with strong basicity^14^, and alkalides with negatively charged alkali metals have been discussed in the literature^12,15^. Similarly, superalkali and superhalogen species have been used to construct perovskites for photovoltaics^16,17^, lithium superionic conductors^18^, etc. Experimentally, a series of superatomic solids assembled from molecules, like nickel telluride clusters and fullerenes^19^, have been successfully synthesized^20^, and interesting properties like controllable phase transitions have been studied^21–24^. However, the reports on supersalts composed of both superalkali and superhalogen ions are still scarce. For example, in the supersalt Na_3_OBH_4_ the superalkali Na_3_O has tetrahedral geometry while it is planar when held in isolation^25^.

The bonding between adjacent superalkali cations and superhalogen anions in supersalts is ionic. Generally, ionic solids adopt structures with higher coordination numbers to maximize the ionic bonding, increase the Madelung constant, and, hence, reduce the lattice energy. As a result, typical structures of ionic binary systems like alkali halides are nonpolar rock salt (RS) or cesium chloride (CsCl) types where the coordination numbers are, respectively, 6 and 8. Due to directionless ionic bonding interactions, they all are nonpolar centro-symmetrical structures. In contrast, the directionality and saturation of covalent bonding could favor the formation of crystalline polarization. According to the Philips scale, when the ionicity declines below the critical value 0.785, marking the idealized boundary between predominantly “covalent” and “ionic” systems^26^, zinc-blende (ZB) structure with lower coordination will become energetically more favorable, e.g., BN (0.256), AlP (0.307), ZnS (0.623), etc. Those structures are polar, although their polarizations are not switchable due to the brittle nature of covalent bonding. Mixed ionic-covalent bonding may facilitate the formation of switchable polarization with moderate barriers (i.e., ferroelectricity), which has been revealed in well-known perovskite ferroelectrics^27^ (BaTiO_3_, PbTiO_3_, etc.). However, we note that supersalts may not necessarily adopt the nonpolar structures for most ionic systems: compared with isotropic single atoms, the anisotropic geometry of superatoms may give rise to breaking of centro-symmetry. If such breaking can induce switchable polarization, highly ionic ferroelectrics might be formed via facile and solution-processed^28^ fabrications, offering a route for large-scale production that cannot be applied to conventional ferroelectrics. This, in turn, can find promising applications in a broad range of fields including nonvolatile memories, sensors and actuators, nonlinear optical materials, etc.

In this paper, using ab initio methods, we design a series of stable supersalts PnH_4_MX_4_ (Pn = N, P; M = B, Al, Fe; X = Cl, Br) composed of superalkali, PnH_4_ and superhalogen, MX_4_, which may enable facile fabrication by exothermal reactions MPn+4HX → PnH_4_MX_4_ or PnH_4_X + MX_3_ → PnH_4_MX_4_. It is noteworthy that the synthesis of similar compounds like NH_4_BCl_4_^29^, NH_4_GaCl_4_^30^, NH_4_InCl_4_^31^, NH_4_AlCl_4_^32^, NH_4_FeCl_4_^33^, NH_4_FeBr_4_^34^, have already be reported decades ago. These ionic systems are likely to form intriguing distorted ZB structure, instead of nonpolar centro-symmetrical structure. The covalent-like directionality gives rise to unprecedented ferroelectricity with long ion displacement (~3 Å) and ferroelasticity with large reversible strain (>40%), which are multiferroically coupled^35^. Conventional ferroelectrics and ferroelastics, owing to the brittle nature of covalent bonding, cannot sustain such large deformations as the high-energy barriers may induce fracture. In these ionic supersalts, however, the long-range Coulomb interaction between cations and anions may greatly reduce such barriers to ultra-low magnitude of only ~0.1 eV/f.u. or ~10 meV/atom. It is noteworthy that the experimental realization of multiferroics with two coupling ferroics (biferroics) is still challenging due to the mutual exclusive origins of different ferroics^36^. Conventional multiferroics, which are mostly metal oxides, can be classified into type-I where ferroelectricity and magnetism arise from different mechanisms, and type-II where ferroelectricity is induced by magnetic order. The multiferroic couplings in type-I and the FE polarization in type-II (<0.1 µC/cm^2^) are weak, which impede their practical applications. Herein, PH_4_FeBr_4_ is even found to be intrinsically triferroic with robust and coupled ferroelectricity (>10 µC/cm^2^), ferroelasticity, and antiferromagnetism, which are neither type-I nor type-II, where the spin directions can be altered via either ferroelastic switching or 90-degree ferroelectric switching. This, in turn, can render desirable giant anisotropic magnetoresistance for nonvolatile memories that can be controlled by either electric field or strain.

## Results

### Structure search

**Fig. 1:**
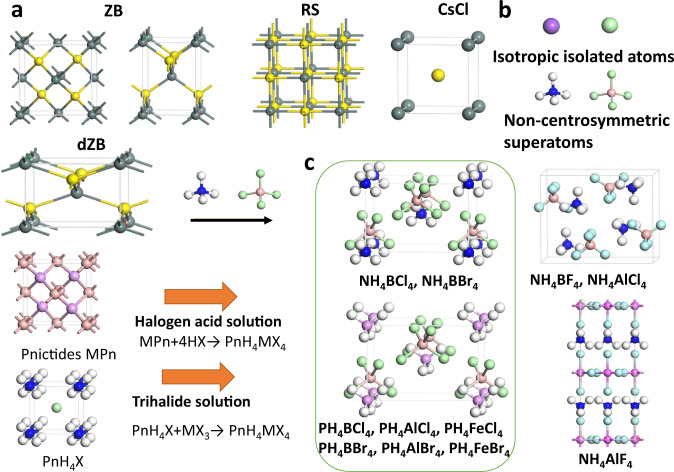
Design of supersalts PnH_4_MX_4_. **a** Structures of ZB, dZB, RS, and CsCl phase, where dark green and yellow spheres, respectively, denote cations and anions. **b** Non-centro-symmetric superalkali and superhalogen compared with isotropic isolated atoms. **c** Several typical structures of supersalts PnH_4_MX_4_, which may be synthesized by reactions MPn+4HX → PnH_4_MX_4_ or PnH_4_X + MX_3_ → PnH_4_MX_4_. White, pink, blue, purple, and light green spheres denote H, B, N, P and halogen atoms, respectively.

The model of ZB, RS, and CsCl structures are displayed in Fig. 1a, where the ZB lattice can either adopt an 8-atom cubic cell (left) or a smaller 4-atom unit cell (right). First, we select superalkali NH_4_ and superhalogen BCl_4_ for constructing a supersalt NH_4_BCl_4_, where both superatoms are non-centrosymmetric compared with isotropic single atoms, as shown in Fig. 1b. After an extensive structure search using the CALYPSO code, we obtain a series of low-energy structures listed in Table S1. The ground state structure of supersalt NH_4_BCl_4_ displayed in Fig. 1c is non-centrosymmetric with independent superalkali NH_4_ and superhalogen BCl_4_ ions. It is a distorted zinc-blende (dZB) structure where the lattice constants (|a|:|b|:|c| = 1.46:1.46:1) are relatively doubled in |a| and |b| (or contracted by half in |c|) compared with the 4-atom unit cell of standard ZB (|a|:|b|:|c|=1:1:1.37). This structure is 0.039 and 0.745 eV/f.u. lower in energy compared with corresponding RS and CsCl structures, respectively. To exclude other polymorphic structures during the synthesis of the ground state dZB phase, temperature and pressure can be controlled, making use of the difference in entropy and density. Choosing proper substrate with perfect lattice match may also facilitate the growth of certain phases while excluding others.

Similar to ZB phase, each superalkali cation in the dZB supersalt is tetrahedrally bonded with four adjacent superhalogen anions and vice versa, akin to *sp*^3^ hybridization of covalent bonding, which are also connected by weak H…Cl hydrogen bonds (with bond length ~2.4 Å). The energy release computed for a hypothetical reaction BN + 4HCl → NH_4_BCl_4_, is as high as 1.10 eV/f.u. (i.e., Δ*E* = −1.10 eV/f.u.), which may enable its solution-processed synthesis via etching of boron nitride by acid. We also examined the following reactions:

1 $${\mathrm{NH}}_4{\mathrm{BCl}}_4 \to {\mathrm{NH}}_4{\mathrm{Cl}} + {\mathrm{BCl}}_3,\,{\Delta} E = 0.14\,{\mathrm{eV}}/{\mathrm{f}}.{\mathrm{u}}.,$$

2 $${\mathrm{NH}}_4{\mathrm{BCl}}_4 \to {\mathrm{NH}}_3{\mathrm{BCl}}_3 + {\mathrm{HCl}},\,{\Delta} E = 0.41\,{\mathrm{eV}}/{\mathrm{f}}.{\mathrm{u}}.,$$

These hypothetical decompositions are all endothermal, which confirm the chemical stability of NH_4_BCl_4_ and impedes the formation of other products like NH_4_Cl or NH_3_BCl_3_ during the synthesis. However, the strong covalent B–N bonding of BN may lead to high-energy cost to break those bonds, so we cannot guarantee that the formation BN + 4HCl → NH_4_BCl_4_ would spontaneously proceed at ambient conditions. On the other hand, the reaction NH_4_Cl + BCl_3_ → NH_4_BCl_4_ might be a more feasible route for the fabrication of NH_4_BCl_4_. For the same group pnictides like BP, AlN, the bonding is weaker. The larger hollow space may enable protons or halogen anions to permeate from the surface to inside, which should facilitate similar formations of supersalts. Hence, we further searched for a series of low-energy structures for NH_4_MX_4_/PH_4_MX_4_ supersalts, as listed in Table S1. As shown in Fig. 1c, the ground state of NH_4_BBr_4_ shares the same type of dZB structure with NH_4_BCl_4_, which is similar to the case for PH_4_BCl_4_, PH_4_AlCl_4_, PH_4_BBr_4_, PH_4_AlBr_4_, with a slight difference in the relative angles between the cation tetrahedrons and anion tetrahedrons. The 3d metal like Fe may also be used to construct superhalogen like FeCl_4_, and the corresponding supersalts like PH_4_FeCl_4_ and PH_4_FeBr_4_ turn out to possess similar dZB ground state structures. Their formations are all energetically favorable, with positive energy releases as listed in Table 1. There are a few exceptions like NH_4_BF_4_ and NH_4_AlCl_4_ that prefer to form structures where each superalkali cation is hydrogen-bonded to eight adjacent superhalogen anions and vice versa, while NH_4_AlF_4_ forms a layered perovskite structure, as shown in the right panel of Fig. 1c.

**Table 1:** The polarization of PnH_4_MX_4_ and the energy change for the reaction PnH_4_X + MX_3_ → PnH_4_MX_4_, where negative values indicate exothermal.

	NH_4_BCl_4_	NH_4_BBr_4_	PH_4_BCl_4_	PH_4_AlCl_4_	PH_4_FeCl_4_	PH_4_BBr_4_	PH_4_AlBr_4_	PH_4_FeBr_4_
Δ*E* (eV/f.u.)	−0.14	−0.05	−0.23	−0.55	−0.63	−0.12	−0.48	−0.89
P(µC/cm^2^)	13.8	12.4	13.1	11.7	11.7	11.8	10.6	10.7

### Ferroelectricity

**Fig. 2:**
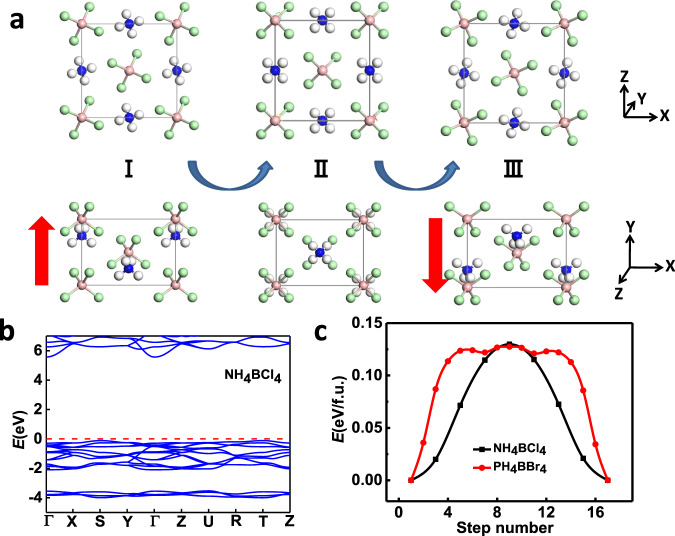
Ferroelectricity. **a** Illustration of ferroelectric switching pathway for NH_4_BCl_4_ and **b** its band structure. **c** Comparison of ferroelectric switching pathway between NH_4_BCl_4_ and PH_4_BBr_4_. The polarization directions are denoted by the red arrows.

Next, we focus on the polar dZB structures in Fig. 1c, which could be ferroelectric as long as their polarizations are switchable. In previous studies^37^, the computed switching barriers for ZB structures like CuCl and ZnO can be over 0.30 eV/f.u., so their polarizations are difficult to reverse under ambient conditions. Compared to the breaking of covalent bonds in those structures during polarization switching, the energy barrier for ion displacement in ionic supersalts should be much lower. Taking NH_4_BCl_4_ shown in Fig. 2a as the paradigmatic case, if all the NH_4_ cations at the initial ground state (I) are simultaneously displaced by 2.63 Å along –y direction, with the rotation of all superatoms by a certain angle along –y axis, an equivalent structure (III) with a reversed polarization can be obtained. The reversed polarization turns out to be of a considerable value, namely, 13.8 µC/cm^2^ as listed in Table 1. Its band structure in Fig. 2b reveals that the system is insulating with a large bandgap over 5 eV, which should result in high dielectric breakdown voltage. The ferroelectric switching pathway is computed by SSNEB calculations in Fig. 2c, with a symmetric state as the intermediate state (II), revealing a switchable barrier of only 0.13 eV/f.u. (0.013 eV/atom). Such a high ion displacement with such a small switching barrier is unimaginable in traditional ferroelectrics. For example, the ion displacement in PbTiO_3_ during ferroelectric switching is below 1 Å, while the barrier is even larger (~0.15 eV/f.u., i.e., 0.03 eV/atom)^38^.

**Fig. 3:**
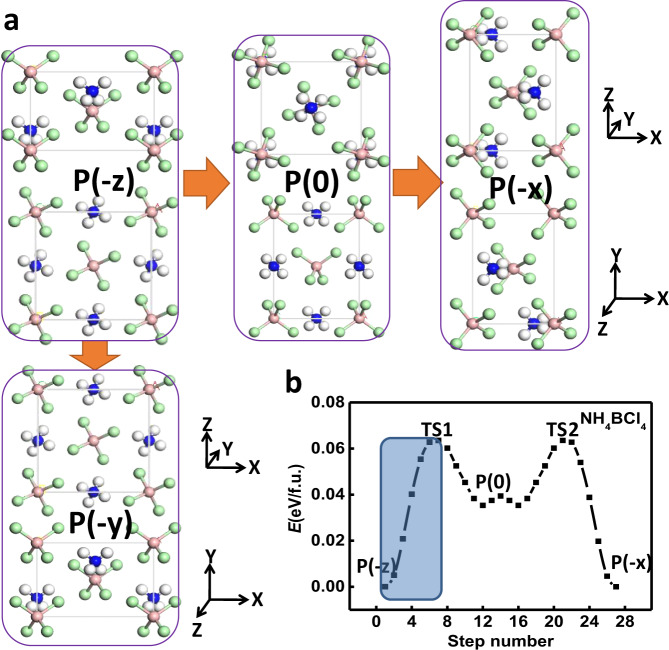
Ferroelasticity. **a** Ferroelastic switching for NH_4_BCl_4_ with its polarization initially along –z direction switched to either –x or –y direction. **b** Computed ferroelastic switching pathway via SSNEB.

The dynamical stability of the polar ground state for NH_4_BCl_4_ is further verified by the phonon spectra in Fig. S1, which shows no imaginary frequencies in all vibration spectra. For the nonpolar intermediate state II (paraelectric phase), the imaginary soft optical modes will lead to both rotation and translational displacement of NH_4_ cations and BCl_4_ anions away from the centrosymmetric position, giving rise to the spontaneous symmetry-breaking below the Curie temperature. Born–Oppenheimer molecular dynamics simulations are also performed to check its thermal stability. Snapshots of the equilibrium structure for NH_4_BCl_4_ at 300 K and at the end of 5 ps are shown in Fig. S2, suggesting the robustness of its ferroelectricity at ambient conditions. Similar mechanism of ferroelectric switching can be applied to other supersalts like PH_4_BBr_4_ via the displacement of cations and anions (2.86 Å along –y direction). The switching barrier in this case is also around 0.13 eV/f.u. (i.e., 0.013 eV/atom, as shown in Fig. 3c). The only difference is the rotational mode of superatoms during ferroelectric switching, as displayed in Fig. S3.

### Ferroelasticity

We note that the dZB structure is relatively elongated in two directions, i.e., contracted in the third direction compared with standard ZB phase, which is actually a spontaneous strain that might be switched via stress. The pathway for such ferroelastic switching for NH_4_BCl_4_ is calculated by SSNEB method, where a symmetrical nonpolar state P(0) can be the intermediate state as its polarization switched from –z direction at the initial P(−z) state to –x/–y direction at the final state P(−x)/P(−y), as shown in Fig. 3a, b. Here, the superiority of ionic ferroelastics with long-range Coulomb interactions between ions should be highlighted, as a barrier of only 0.065 eV/f.u. (i.e., 0.0065 eV/atom) is required for reversing such a high ferroelastic strain (defined as (|b|/|c|−1) × 100%) more than 40%. For high-strain (>20%) ferroelastics predicted in previous reports^39–42^, the switching barriers (up to 0.5 eV/atom) almost orders of magnitude higher may induce fracture, which can be attributed to the distinct features of covalent bonds in those systems. As revealed by the pathway in Fig. 3c, during the ferroelastic switching, the structure of P(−z) will be transformed into transition state TS1 that is 0.065 eV higher in energy, and then automatically transformed into metastable phase with lower energy, and symmetrically to another high-energy transition state TS2 followed by another equivalent ground state P(−x) or P(−y). Our calculated stress–strain curve in Fig. S2(c) shows that the highest stress during ferroelastic switching is below 1 GPa (close to the stress induced by a small tensile strain of only 1% applied on zinc-blende ZnS). A snapshot of this point with the highest stress at 300 K and at the end of 5 ps indicates that fracture should not take place during switching. Moreover, the ferroelectric polarization is also rotated by 90 degree upon ferroelastic switching. Such 90-degree polarization switching can also be achieved if an external electric field is applied along –x or –y direction. Hence, the ferroelectricity and ferroelasticity are coupled, so we can use strain to control the polarization direction and electric field to control strain. Due to the low barrier, the required electric field or stress for reversing such a large strain can be greatly reduced. It is noteworthy that a tensile strain is usually applied by clamping on the surface of a material, which is likely to induce non-uniformity, facilitating fracture. In contrast, the electrical force applied on those ferroelectric and ferroelastic supersalts will be much more uniform.

### Triferroicity

**Fig. 4:**
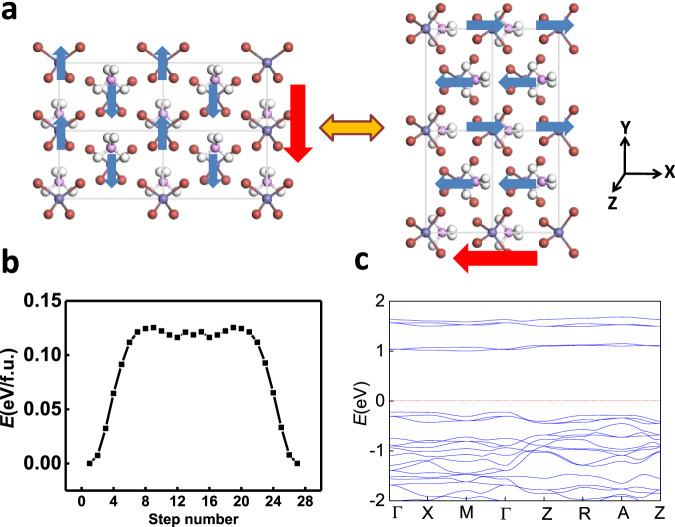
Triferroic coupling. **a** Spin switching upon ferroelastic switching (90-degree ferroelectric switching), **b** ferroelastic switching pathway calculated by SSNEB method, and **c** the band structure for PH_4_FeBr_4_. Red and blue arrows denote polarization and spin directions, respectively.

Supersalts with 3d magnetic ions like PH_4_FeBr_4_ also possess similar coupled ferroelectricity and ferroelasticity. Moreover, their magnetism can also be coupled. We have checked different possible spin configurations (see Table S1) for PH_4_FeBr_4_. The ground state is displayed in Fig. 4a, which is antiferromagnetic where the spins of two Fe ions in the unit cell are in the opposite direction, while the spins on the same lines along –x, –y or –z axes are in the same direction. Further, noncollinear spin calculations reveal a magnetic anisotropy energy of 0.4 meV for the spin of each Fe atom, where the spins are preferably aligned along –y direction, the “contracted” direction in Fig. 4a. However, as the “contracted” direction is switched to –x direction upon ferroelastic switching, i.e., 90-degree ferroelectric switching, the magnetization easy axis is also switched from –y to –x direction. Equivalently, it might also be switched to –z direction via a strain or electric field along –z direction. Compared with NH_4_BCl_4_, the switching barrier of PH_4_FeBr_4_, according to the calculated pathway in Fig. 4b, is larger (~0.13 eV/f.u.), while the bandgap is much more reduced (~1 eV, see Fig. 4c) to a desirable range for nanoelectronics. Hence, magnetism is also coupled with ferroelectricity and ferroelasticity in PH_4_FeBr_4_, which can be viewed as a multiferroic material with “triferroic” coupling. Such coupling may render electrically controlled giant anisotropic magnetoresistance for nonvolatile memories, which is highly desirable but still challenging in the current developing field of antiferromagnetic spintroincs^43^.

## Discussion

In summary, using an unbiased structure search based on DFT calculations, we predict a series of stable ionic supersalts PnH_4_MX_4_ (Pn = N, P; M = B, Al, Fe; X = Cl, Br) composed of superalkali PnH_4_ and superhalogen MX_4_ ions. The reactions MPn+4HX → PnH_4_MX_4_ or PnH_4_X + MX_3_ → PnH_4_MX_4_, which are all exothermic, may render facile and large-scale fabrication of these super-ionic salts. These supersalts form intriguing distorted ZB structures with covalent-like directional bonding. This unique property is attributed to the anisotropy induced by the superatoms. The special configuration enables ferroelectricity with ultra-long ion displacement, as well as ferroelasticity with ultra-large reversible strain. Coupled together, one can realize strain-controllable polarization as well as electrically controllable strain. The ionic bonding features reduce the switching barriers with large deformations to only around ~0.1 eV/f.u.(~10 meV/atom). This is inconceivable in conventional ferroelectrics and ferroelastics because it cannot be sustained due to the brittle nature of covalent bonding. Finally, we show that PH_4_FeBr_4_ is triferroics with coupled ferroelectricity, ferroelastcity, and antiferromagnetism, where the spin distribution can be altered via either ferroelastic switching or 90-degree ferroelectric switching. Since supersalts and triferroics are both long-sought but still elusive to date, our findings offer new ideas to stimulate experimental efforts in these fields.

## Methods

Our theoretical calculations are based on density-functional-theory (DFT) methodologys implemented in the Vienna Ab initio Simulation Package (VASP 5.3) code^44,45^. The Perdew–Burke–Ernzerhof^46^ functional within the generalized gradient approximation for the exchange and correlation potential, together with the projector-augmented wave^47^ method, are adopted. The kinetic energy cut-off is set to 520 eV in all calculations. The Brillouin zones are sampled by Monkhorst−Pack scheme^48^, which is set to 5 × 7 × 5 for the unit cell. The shape and volume of each unit cell are fully optimized, and the convergence threshold for self-consistent-field iteration is set to 10^−6^ eV. Computed forces at all atomic sites are less than 0.01 eV/Å after geometry optimization. The van der Waals interactions are taken into consideration by using DFT-D3 functional of Grimme^49^. The Berry phase method^50^ is employed to evaluate ferroelectric polarizations, while the ferroelectric/ferroelastic switching pathway is obtained by using a generalized solid-state elastic band (G-SSNEB) method^51^. The phonon calculations are performed using the finite displacement method as implemented in the PHONOPY program^52^. An unbiased swarm-intelligence structural method based on the particle swarm optimization (PSO) technique implemented in the Crystal structure AnaLYsis by Particle Swarm Optimization (CALYPSO) code^53–55^ is employed to search for low-energy structures of supersalts. For the PSO algorithm, the behavior of each individual is affected by either the best local or the best global individual to help it fly through the hyperspace. Moreover, an individual can learn from its past experiences to adjust its flying speed and direction, so all the individuals in the swarm can quickly converge to the global position. All 230 space groups are allowed for generation of structures, and 60% of the low-energy structures in previous generation are produced by PSO, while the rest are randomly generated with symmetry constraints.

## Supplementary information

Supplementary Information

## Data Availability

The authors declare that the main data supporting the findings of this study are contained within the paper and its associated Supplementary Information. All other relevant data are available from the corresponding author upon request.

## Code availability

Calypso code is free for academic use online: www.calypso.cn. All other relevant scripts are available from the corresponding author upon request.
