# Supplementary material for: A family of ionic supersalts with covalent-like directionality and unconventional multiferroicity
Source: Nat Commun. 2021 Feb 26;12:1331. doi: 10.1038/s41467-021-21597-3 (PMC7910577; doi:10.1038/s41467-021-21597-3)
Supplement: Supplementary file 1 — Supplementary Information [file 41467_2021_21597_MOESM1_ESM.pdf]

Supplementary Information

A Family of Ionic Supersalts with Covalent-like Directionality and Unconventional Multiferroicity

Yaxin Gao[1], Menghao Wu[1]*, Puru Jena[2]

[1]School of Physics and Wuhan National High Magnetic Field Center, Huazhong University of Science and Technology, Wuhan, Hubei 430074, China

[2]Department of Physics, Virginia Commonwealth University, Richmond, Virginia 23284, USA

Table S1. Relative energies (eV/f.u) of some low-energy phases compared with the ground state, searched by using CALYPSO code where 19 generations (30 structures per generation) were generated for each supersalt.

	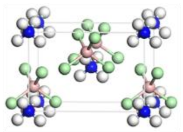	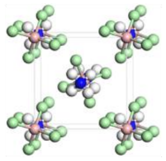	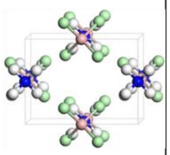	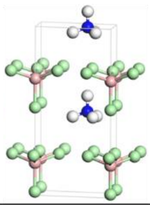	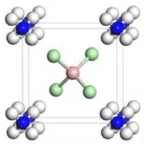	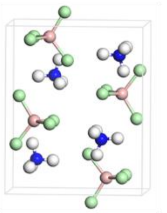	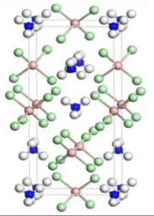
**NH_4BCl_4**	**0**	**0.039**	**0.105**	**0.151**	**0.745**	**0.047**	**0.240**
**NH_4BBr_4**	**0**	**0.070**	**0.085**	**0.179**	**0.908**	**0.070**	**0.350**
**PH_4BCl_4**	**0**	**0.079**	**0.232**	**0.188**	**0.476**	**0.087**	**0.188**
**PH_4AlCl_4**	**0**	**0.080**	**0.131**	**0.281**	**0.423**	**0.049**	**0.110**
**PH_4FeCl_4**	**0**	**0.098**	**0.144**	**0.310**	**0.418**	**0.096**	**0.117**
**PH_4BBr_4**	**0**	**0.130**	**0.230**	**0.243**	**0.654**	**0.111**	**0.260**
**PH_4AlBr_4**	**0**	**0.099**	**0.131**	**0.262**	**0.592**	**0.087**	**0.150**
**PH_4FeBr_4**	**0**	**0.103**	**0.153**	**0.385**	**0.553**	**0.130**	**0.146**
**NH_4BF_4**	**0.163**	**0.084**	**0.247**	**0.211**	**0.506**	**0**	**0.057**
**NH_4AlCl_4**	**0.087**	**0.016**	**0.074**	**0.268**	**0.724**	**0**	**0.085**
**NH_4InCl_4**	**0.099**	**0.059**	**0.096**	**0.345**	**0.880**	**0.092**	**0**

	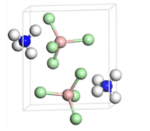	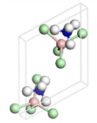	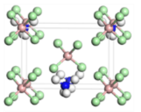	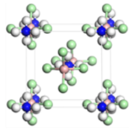	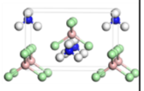
**NH_4BCl_4**	**0.056**	**0.119**	**0.119**	**0.142**	**0.216**

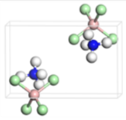	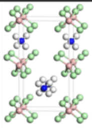	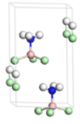	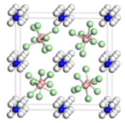	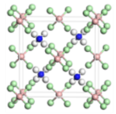	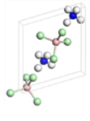	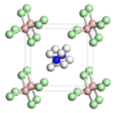	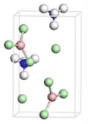
**0.120**	**0.295**	**0.413**	**0.435**	**0.436**	**0.448**	**0.462**	**0.486**

	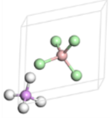	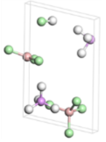	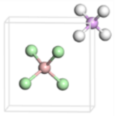	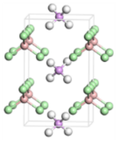	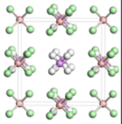
**PH_4BCl_4**	**0.186**	**0.425**	**0.457**	**0.561**	**0.789**

	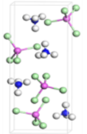	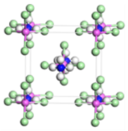	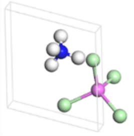
**NH_4AlCl_4**	**0.025**	**0.123**	**0.127**

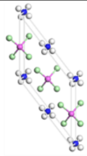	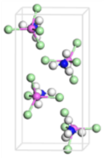	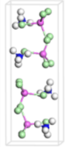	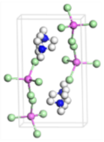	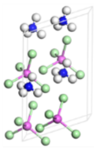	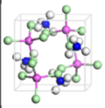	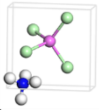	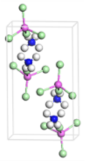
**0.057**	**0.077**	**0.116**	**0.137**	**0.138**	**0.161**	**0.190**	**0.196**

	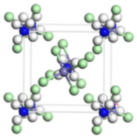	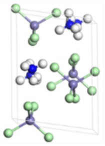	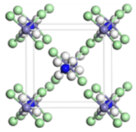	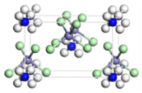	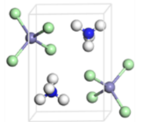	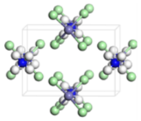	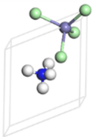
**NH_4FeCl_4**	**0**	**0.005**	**0.050**	**0.058**	**0.063**	**0.065**	**0.177**

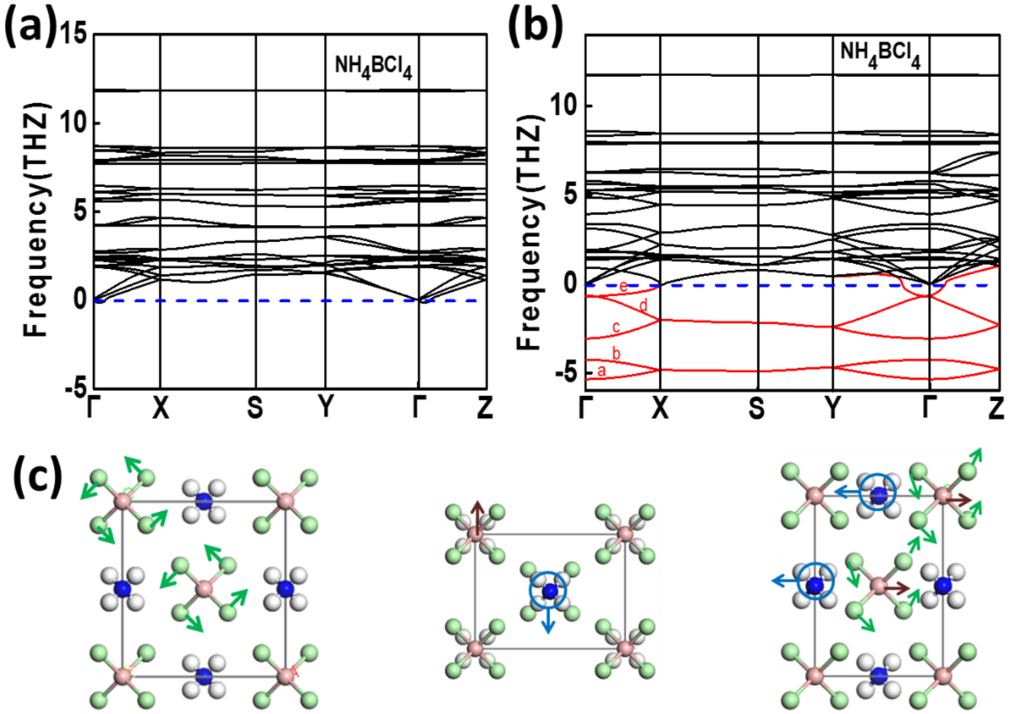

Figure S1. Phonon spectra of NH_4BCl_4 for (a)the ground state polar phase and (b)the paraelectric phase. (c)corresponds to mode d inducing both rotation and translational displacement of NH_4 cations and BCl_4 anions away from the centrosymmetric position.

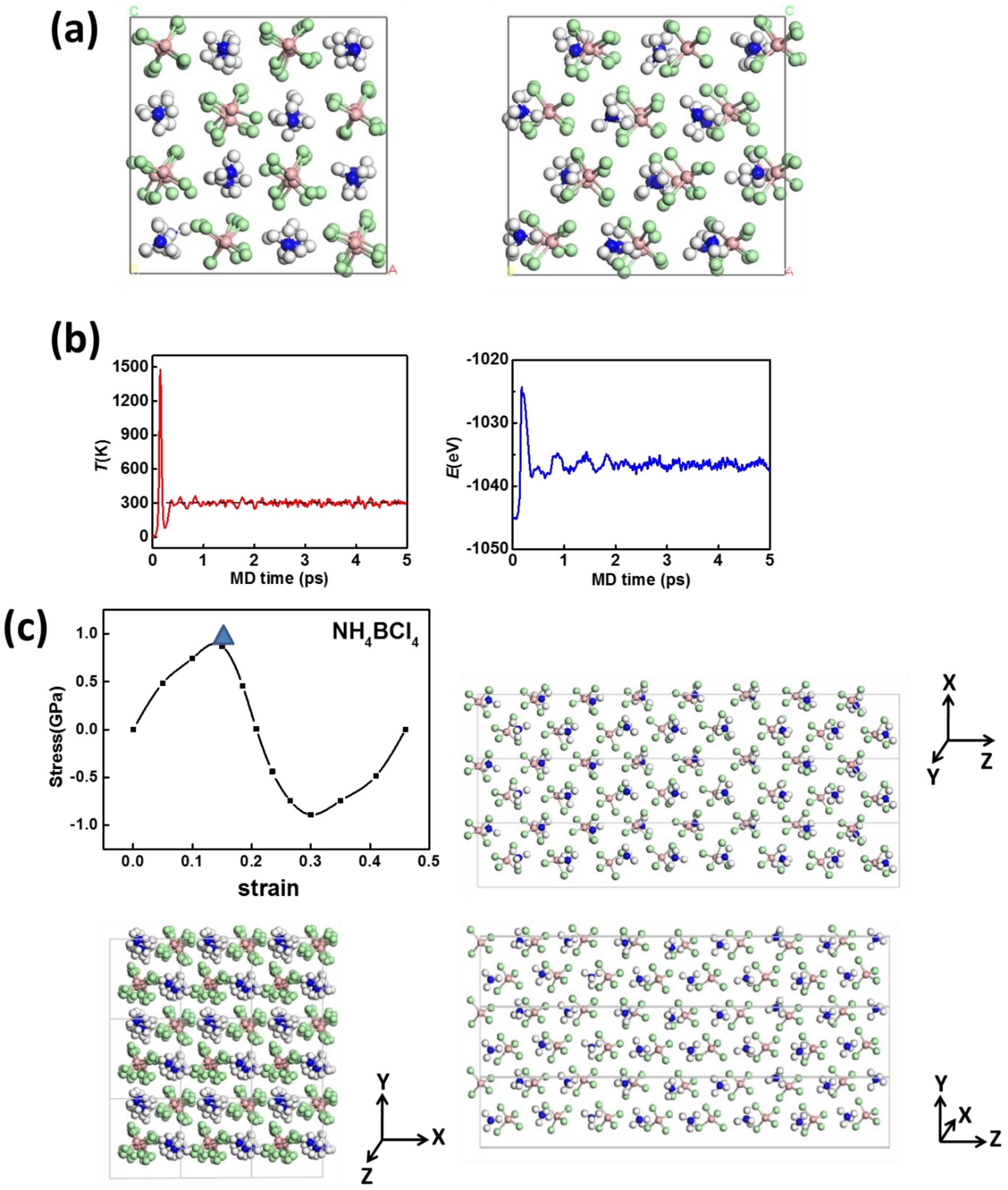

Figure S2. (a) A snapshot of the equilibrium structure (over-view and side-view) for NH_4BCl_4 at 300 K and at the end of 5 ps of BOMD simulation. (b)The evolution of temperature and energy during the MD simulation. (c) Stress-strain curve of NH_4BCl_4 and a snapshot of the marked point with the highest stress at 300 K and at the end of 5 ps of BOMD simulation, where a 1 × 1 × 8 supercell is adopted and the strain is applied along –z axis.

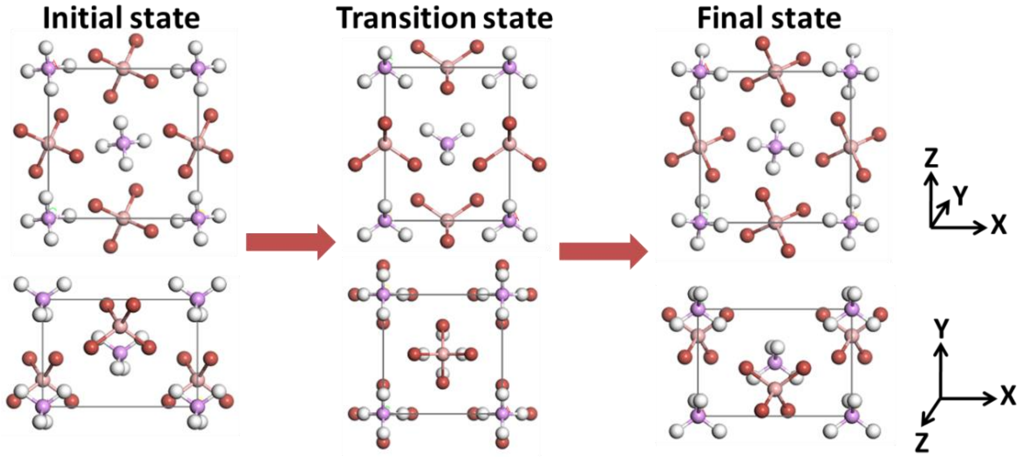

Figure S3. Illustration of ferroelectric switching pathway for PH_4BBr_4.

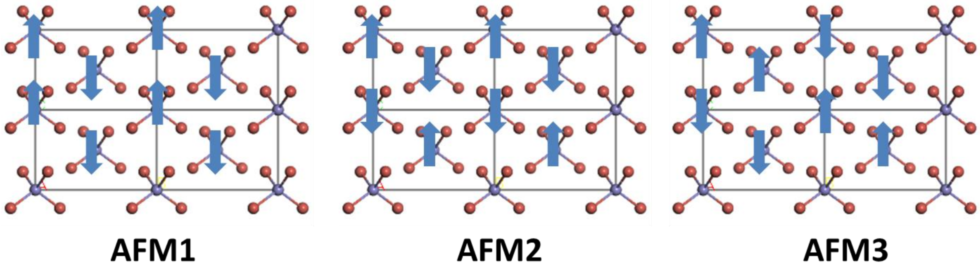

	FM	AFM1	AFM2	AFM3
Δ*E*(meV/f.u.)	0	-20.0	-12.5	-11.3

Table S2. Relative energy of different spin configurations compared with ferromagnetic state.
